# Influence of false beliefs and empathy on white lies among children with mild intellectual disabilities: focusing on trait and state perspectives

**DOI:** 10.3389/fpsyg.2024.1502606

**Published:** 2024-11-11

**Authors:** Zeng Zhen, Meng Liming

**Affiliations:** ^1^School of Education Science, Huaihua University, Huaihua, China; ^2^Wulingshan K-12 Educational Research Center, Huaihua University, Huaihua, China; ^3^Special Education School, Jining, China

**Keywords:** children with mild intellectual disabilities, false belief, empathy, white lie, social adaptation, educational interventions

## Abstract

Children with mild intellectual disabilities often exhibit poor social skills owing to intellectual impairments. This makes it essential to enhance their communication abilities. This study provides a novel contribution by systematically investigating the effects of false beliefs and empathy on white lie behavior among children with mild intellectual disabilities, considering both state and trait dimensions. Experiment 1 examined the impact of trait-level false beliefs and trait-level empathy on trait-level white lies. The results demonstrated that trait-level false beliefs and trait-level empathy both significantly promoted white lie behavior. Experiment 2 explored the influence of state-level false beliefs and state-level empathy on state-level white lies and found similar positive effects. By integrating both trait and state perspectives, this research fills a gap in the literature on white lie behavior in children with mild intellectual disabilities and uncovers the mechanisms through which false beliefs and empathy operate in different contexts. These findings offer comprehensive educational and intervention strategies to improve social adaptation in children with mild intellectual disabilities.

## 1 Introduction

Children with intellectual disabilities, also known as children with intellectual impairment or children with intellectual delay, are children whose intelligence is significantly lower than the average and who display poor adaptive behavior (Larson et al., 2001). Children with mild intellectual disability have an IQ ranging from 50 to 69, along with deficient adaptive function (Schalock et al., 2021). Owing to their intellectual deficiency, these children often express friendliness inappropriately, which leads to poor social relationships (Webster and Carter, 2013). This makes it essential to foster their social interactions with the general population and help them establish and maintain positive relationships. Modern special education focuses on promoting their interaction with the general population, helping them build strong relationships, and facilitating their social adaptability (Kanner et al., 1972).

A white lie refers to a deliberate falsehood one tells to avoid causing negative emotional experiences or to protect others' interests (Bok, 1978; Erat and Gneezy, 2012; Talwar et al., 2007). Research on children's white lie behavior has primarily focused on typically developing children. Talwar and Lee (2002) found that typically developing children as young as three years can tell white lies, and other researchers have reached similar conclusions (Broomfield et al., 2002; Bussey, 1999; Popliger et al., 2011; Thompson and Newton, 2013). Chinese scholars have shown that the ability to tell white lies among typically developing children aged 4 to 6 years is closely related to time; it develops as their physical and psychological functions mature. Studies have also revealed that typically developing children are more willing to say untruthful things to maintain friendships (Ma et al., 2011), and prosocial and self-protection motivations are the main drivers of this behavior (DePaulo et al., 2004; Glätzle-Rützler and Lergetporer, 2015).

Research on white lie behavior among children with intellectual disabilities is relatively scarce. Existing studies have suggested that such children face challenges in understanding and using white lies but exhibit some capacity for it (Thirion-Marissiaux and Nader- Grosbois, 2008). Studies on children with hearing impairments and other special needs have shown that their white lie behavior differs from that of typically developing children; it is influenced by their specific needs and environment (Hao and Wu, 2019). These findings highlight the necessity of this study, as it explores white lie behavior among children with mild intellectual disabilities and the factors that influence it. This endeavor can provide theoretical support for improving their social adaptability.

Studies have shown that false beliefs affect white lie behavior among typically developing individuals. However, few studies have examined this effect among children with mild intellectual disabilities (Bara et al., 2001; Happé, 1995; Sodian and Frith, 1992). False beliefs can be of two types: trait-level and state-level false beliefs. Having trait-level false beliefs is being able to understand others' beliefs and intentions consistently. This is a stable psychological characteristic that influences how one interprets others' mental states in different situations (Devine and Hughes, 2018; Wellman et al., 2001). The concept of false beliefs is closely related to theory of mind, which refers to one's capacity to understand that others have their own beliefs, desires, and intentions (Baron-Cohen et al., 1985).

Research has shown that children with intellectual disabilities perform worse than typically developing children in theory of mind tasks. This finding suggests that children with intellectual disabilities exhibit a lasting deficit in understanding others' beliefs and intentions. This deficit may significantly affect their performance of trait-level white lies, which are lies one tells for altruistic motives or to avoid hurting others' feelings (Siriattakul et al., 2021). Studies have shown that trait-level false beliefs predict the likelihood of typically developing individuals telling white lies (Isaksson et al., 2021; Thijssen et al., 2017). This finding raises the question of whether this effect also applies to children with mild intellectual disabilities. Therefore, in this study, Hypothesis 1 proposed that trait-level false beliefs positively predict trait-level white lies among children with mild intellectual disabilities.

Past research has mostly focused on the effect of trait-level false beliefs on white lie behavior, neglecting differences in white lie behavior under different states of false beliefs. When it comes to state-level false beliefs, having weak theory of mind can lead to difficulties in understanding other people's intentions in specific situations. This can significantly affect one's behavior and responses in specific situations. Children with intellectual disabilities often perform poorly on false-belief tasks, which suggests that they may face difficulties in understanding others' mental states. In fact, Baron-Cohen et al. (1985) found that children with intellectual disabilities perform significantly worse than typically developing children on a series of false-belief tasks. This deficiency may affect their performance of state-level white lies, which are lies one tells in specific situations to avoid hurting others' feelings or for other altruistic reasons (Sodian and Frith, 1992). Thus, Hypothesis 2 proposed that state-level false beliefs positively predict state-level white lies among children with mild intellectual disabilities.

Recent research has indicated that false beliefs may not be the only factor influencing white lie behavior. Some studies have shown that empathy plays a crucial role in understanding others' beliefs and intentions and can influence the performance of white lies (Lee et al., 2019). Empathy, which also has trait and state levels, is the ability to understand and share others' emotional states consistently and is critical for the performance of trait-level white lies. Highly empathetic individuals are more likely to use trait-level white lies in different situations to avoid hurting others' feelings (Eisenberg, 2006; Moore et al., 2015). Yirmiya and Sigman (1991) found that children with intellectual disabilities perform poorly on empathy tasks, which suggests that these children face difficulties in understanding and sharing others' emotional states. These difficulties can affect their performance of trait-level white lies. Thus, Hypothesis 3 proposed that trait-level empathy positively predicts trait-level white lies among children with mild intellectual disabilities.

Empathy is also essential for the performance of state-level white lies, as it allows one to understand and share others' emotional states in specific situations (Davis, 1983; Decety and Jackson, 2004). Individuals with high levels of empathy are more likely to use state-level white lies to avoid hurting others' feelings (Eisenberg and Miller, 1987). Sigman et al. (1999) noted that children with intellectual disabilities perform worse on empathy tasks than typically developing children, which could result in poorer state-level white lie performance. Therefore, Hypothesis 4 proposed that state-level empathy positively predicts state-level white lies among children with mild intellectual disabilities.

Existing research has primarily focused on the white lie behavior of typically developing children, whereas such research remains relatively scarce among children with mild intellectual disabilities (Fan et al., 2022). Although some studies have examined the lying behavior of children with intellectual disabilities (Glätzle-Rützler and Lergetporer, 2015), their capacity for white lies remains unclear. Additionally, most studies have focused on state-level variables (e.g., state-level false beliefs and state-level empathy) whose instability and subjectivity limit the generalizability of the findings (Bernstein and Eveland, 1982; Spielberger, 2010).

To address these gaps, this study investigated the influence of false beliefs and empathy on white lies among children with mild intellectual disabilities from both trait and state perspectives. Through behavioral experiments, this study aimed to provide theoretical and practical insight into enhancing the social abilities of children with mild intellectual disabilities.

## 2 Experiment 1: Effect of trait-level false beliefs and trait-level empathy on trait-level white lies among children with mild intellectual disabilities

### 2.1 Objective and hypothesis

Experiment 1 tested Hypotheses 1 and 3. More specifically, it examined the impact of trait-level false beliefs and trait-level empathy on trait-level white lies among children with mild intellectual disabilities and typically developing children. It investigated whether differences in trait-level false beliefs and trait-level empathy between the two groups of children cause differences in their trait-level white lie behavior.

### 2.2 Methods

#### 2.2.1 Participants

The required sample size was calculated using G-power 3.1 (Faul et al., 2007). For an effect size of *f* = 0.5, a power of β = 0.80, and α = 0.05, the required sample size was 102. A total of 165 children (65 children with mild intellectual disabilities and 100 typically developing children) were randomly selected for Experiment 1. Fifty children with mild intellectual disabilities were selected from the Special Education Center in Chengwu County, Heze City, Shandong Province, China, and 15 children with mild intellectual disabilities were selected from the Pediatric Rehabilitation Department of Chengwu County Hospital, Heze City, Shandong Province, China. Among these children, 37 were boys and 28 were girls aged 8-15 years (*M* ± *SD* = 11.38 ± 3.59). Hundred typically developing children were selected from the Second Kindergarten in Yuelu District, Changsha City. They comprised 52 boys and 48 girls aged 3-6 years (*M* ± *SD* = 4.91 ± 0.43). All experiments were conducted in a quiet room at the participants' schools or in the pediatric rehabilitation departments of the respective hospitals. Informed consent was obtained from the parents or guardians of the participants, and the experiments were conducted after obtaining ethical approval from the ethics committee of the unit. The mean score for verbal intelligence was 137.56 (*SD* = 23.33) among children with mild intellectual disabilities and 140.64 (*SD* = 27.95) among typically developing children. The Mann-Whitney U test indicated similar levels of verbal intelligence between the two groups (*z* = −1.52, *p* = 0.10). Table 1 presents the characteristics of the participants.

**Table 1:** Characteristics of the participants.

**Group**	***n***	**Mean age (standard deviation)**	**Age range**	**Mean score for verbal intelligence (standard deviation)**
Children with mild intellectual disabilities	65	11.38 (3.59)	8–15	137.56 (23.33)
Typically developing children	100	4.91 (0.43)	3–6	140.64 (27.95)

#### 2.2.2 Experimental design

This experiment employed a single-factor between-subjects design. The two groups of children (children with mild intellectual disabilities and typically developing children) were the independent variables. The dependent variables were the scores for trait-level white lies, trait-level false beliefs, and trait-level empathy.

#### 2.2.3 Measures

##### 2.2.3.1 Trait-level white lies

Trait-level white lies were assessed using the Child and Adolescent Deception Scale. Lundquist et al. developed the Adult Deception Scale in 2009 to measure deception in adults. Kabha and Berger (2023) adapted this scale for use among children and adolescents and developed the Child and Adolescent Deception Scale. This scale consists of two parts, the Lie Scale and the White Lie Scale, and is divided into subscales for toddlers, children, and adolescents, comprising items such as “I will tell others I did something even if I didn't” and “I will deliberately hide the truth to avoid punishment.” All items are rated on a 5-point Likert scale, with 1 indicating “completely disagree” and 5 indicating “completely agree”. Higher scores indicate more deceptive behavior, and this scale has shown good reliability and validity in multiple studies (Cronbach's alpha = 0.88; Lundquist et al., 2009; Kabha and Berger, 2023).

##### 2.2.3.2 Trait-level false beliefs

Trait-level false beliefs were measured using the Theory of Mind Scale. This scale was proposed by Wellman and Liu et al. in 2004 and is generally used among children. It includes seven subscales: Different Wishes Scale, Different Beliefs Scale, Knowledge and Ignorance Scale, Content False Belief Scale, Explicit False Belief Scale, Belief-Emotion Scale, and Real-Surface Emotion Scale. The Different Wishes Scale measures wish development. The Different Beliefs Scale measures the development of multiple beliefs. The Knowledge and Ignorance Scale measures one's awareness of others' mental states. The Content False Belief Scale and Explicit False Belief Scale primarily measure one's understanding of false beliefs. The Belief-Emotion Scale measures the development of emotions based on beliefs, and the Real-Surface Emotion Scale measures the development and characteristics of emotions based on content false beliefs. We used the Content False Belief Scale and the Explicit False Belief Scale, comprising scenarios such as “Why do you think he did that?” and “What would he think if he didn't know the truth?” The scale uses a 5-point rating system, with 1 indicating “completely incorrect” and 5 indicating “very correct”. Higher scores indicate stronger theory of mind ability. The scale has demonstrated good reliability and validity in multiple studies (Cronbach's alpha = 0.85; Wellman and Liu, 2004).

##### 2.2.3.3 Trait-level empathy

Trait-level empathy was assessed using the Empathy Scale for Children and Adolescents. By conducting studies involving 56 first-grade students, 115 fourth-grade students, and 87 seventh-grade students, Bryant (1982) confirmed the reliability and preliminary construct validity of this scale. This scale is suitable for children and adolescents and consists of 22 items, with 8 items measuring different levels of empathy toward same-sex and opposite-sex individuals. The items vary in difficulty; some are more challenging and suitable for adolescents with more developed cognitive understanding, while others are simpler and more suitable for younger children with less developed comprehension, such as “When I see someone sad, I also feel sad” and “I can understand why someone is angry”. The scale uses a 5-point rating system, with 1 indicating “completely incorrect” and 5 indicating “very correct”. Higher scores indicate stronger empathy. In this study, we selected and adapted items suitable for children, as children with mild intellectual disabilities have a cognitive understanding closer to that of a child than that of an adolescent. This scale has shown good reliability and validity in multiple studies (Cronbach's alpha = 0.90; Bryant, 1982).

#### 2.2.4 Experimental procedure

To administer the scales, the experimenter asked questions verbally. The participant gave verbal responses, and the experimenter scored and recorded the answer. The participants were tested in a random order to avoid order effects. Each scale took approximately 10 minutes to administer, and the total duration of Experiment 1 was approximately 30 minutes.

#### 2.2.5 Data analysis

The Mann-Whitney U test was conducted to compare the verbal intelligence scores of the two groups of children. Independent samples t-tests were performed to compare the scores for trait-level false beliefs, trait-level empathy, and trait-level white lies in the two groups. Spearman's rank correlation analysis was employed to examine the relationship between trait-level false beliefs, trait-level empathy, and trait-level white lies in the two groups. Finally, multiple linear regression analysis was performed to explore the effects of trait-level false beliefs and trait-level empathy on trait-level white lies among children with mild intellectual disabilities and typically developing children. Trait-level false beliefs and trait-level empathy were the independent variables, while trait-level white lies was the dependent variable. All statistical analyses were performed using SPSS software (version 26.0).

### 2.3 Results

#### 2.3.1 Trait-level white lies, trait-level false beliefs, and trait-level empathy among children with mild intellectual disabilities and typically developing children

**Figure 1:**
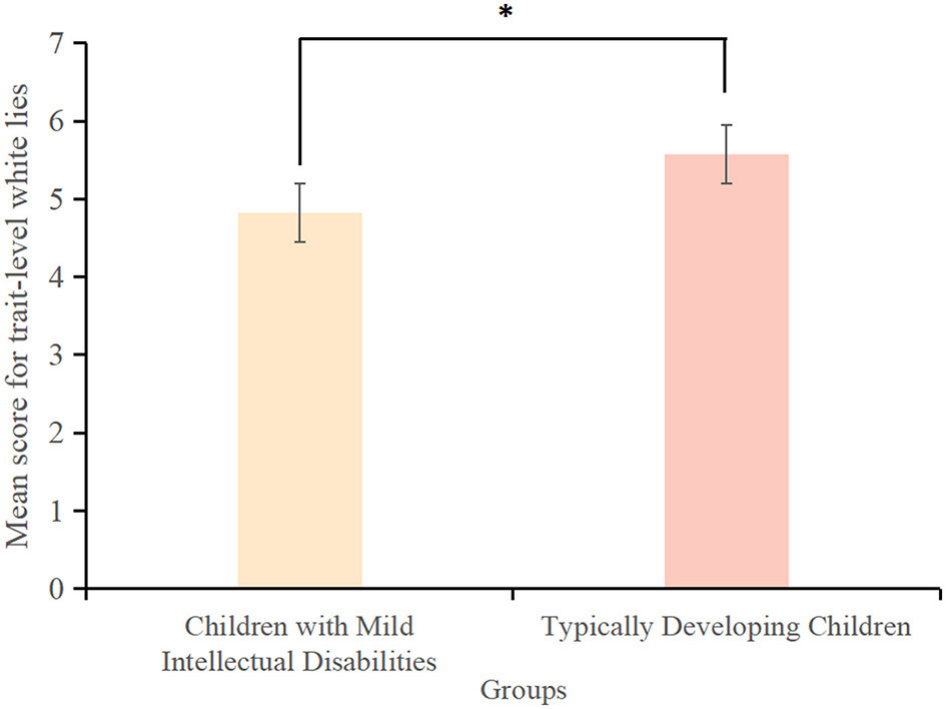
Scores for trait-level white lies among children with mild intellectual disabilities and typically developing children. ^*^p < 0.05.

**Figure 2:**
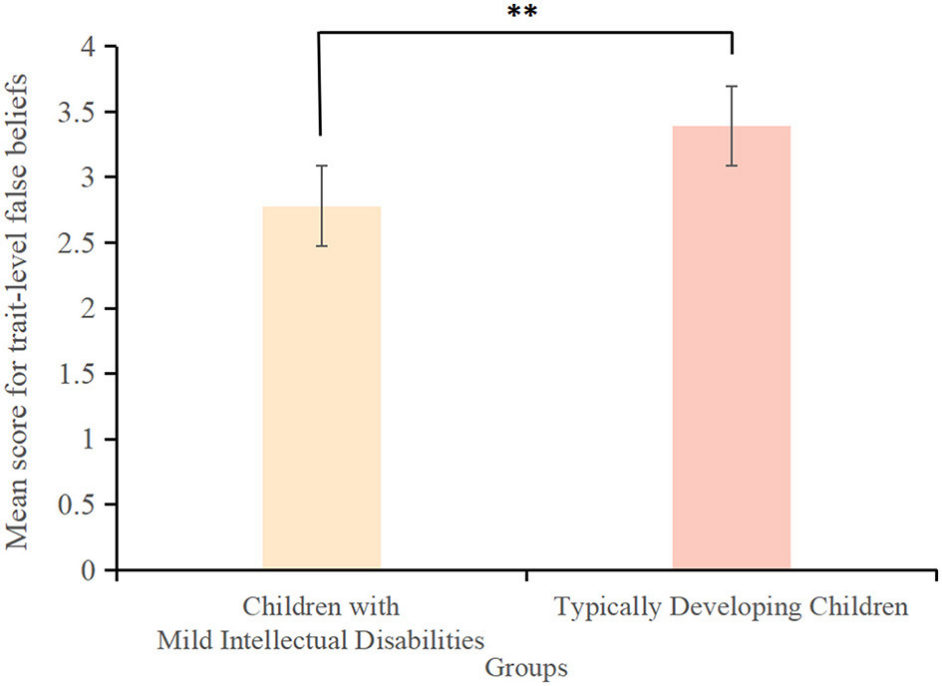
Scores for trait-level false beliefs among children with mild intellectual disabilities and typically developing children. **p < 0.01.

**Figure 3:**
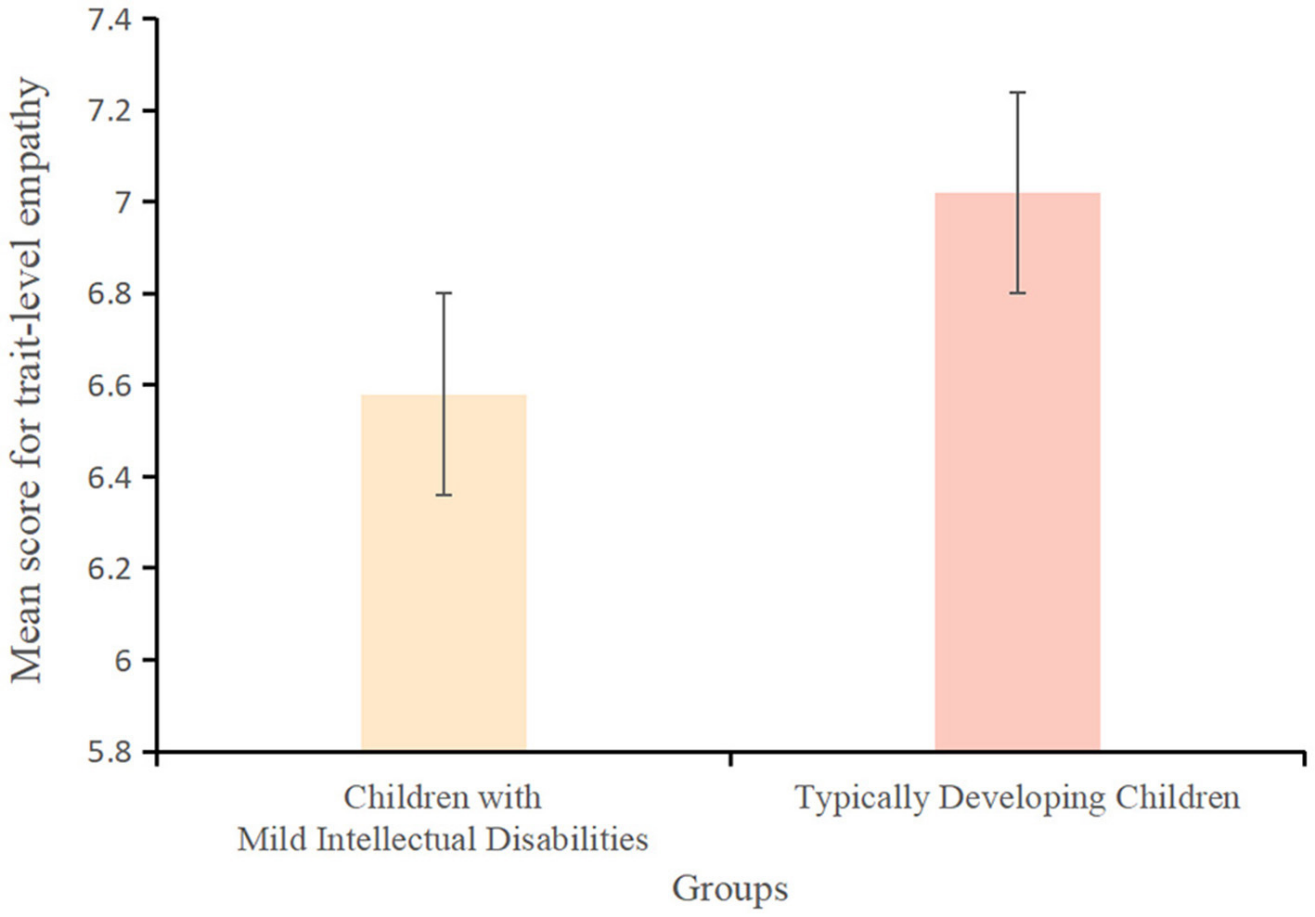
Scores for trait-level empathy among children with mild intellectual disabilities and typically developing children.

The score for trait-level white lies among children with mild intellectual disabilities (4.82 ± 2.48) was significantly lower than that among typically developing children (5.57 ± 2.60) (*t*(163) = −2.32, *p* < 0.001, *Cohen's d* = −0.26, *95% CI* = [0.03, 1.53]) (Figure 1). Similarly, the score for trait-level false beliefs among children with mild intellectual disabilities (2.78 ± 3.39) was significantly lower than that among typically developing children (3.39 ± 0.98) (*t*(163) = −3.52, *p* < 0.001, *Cohen's d* = −0.24, *95% CI* = [-0.37,−0.11]) (Figure 2). However, the score for trait-level empathy did not differ significantly between children with mild intellectual disabilities (6.58 ± 2.84) and typically developing children (7.02 ± 1.88) (*t*(163) = −0.99, *p* > 0.05, *Cohen's d* = −0.19, *95% CI* = [−0.570, 0.190]) (Figure 3).

#### 2.3.2 Correlation between trait-level white lies, trait-level false beliefs, and trait-level empathy among children with mild intellectual disabilities and typically developing children

Among children with mild intellectual disabilities, trait-level white lies were significantly and positively correlated with trait-level false beliefs (*r* = 0.437, *p* < 0.01) and trait-level empathy (*r* = 0.223, *p* < 0.05). Trait-level empathy and trait-level false beliefs were also significantly and positively correlated (*r* = 0.260, *p* < 0.05) (Table 2). Among typically developing children, trait-level white lies were highly positively correlated with trait-level false beliefs (*r* = 0.553, *p* < 0.001). Trait-level empathy was significantly and positively correlated with trait-level white lies (*r* = 0.425, *p* < 0.01) and trait-level false beliefs (*r* = 0.328, *p* < 0.01) (Table 3).

**Table 2:** Results of correlation analysis of trait-level white lies, trait-level false beliefs, and trait-level empathy among children with mild intellectual disabilities.

	**Trait-level white lies**	**Trait-level false beliefs**	**Trait-level empathy**
Trait-level white lies	1.000		
Trait-level false beliefs	0.437^**^	1.000	
Trait-level empathy	0.223^*^	0.260^*^	1.000

^*^p < 0.05, ^**^p < 0.01.

**Table 3:** Results of correlation analysis of trait-level white lies, trait-level false beliefs, and trait-level empathy among typically developing children.

	**Trait-level white lies**	**Trait-level false beliefs**	**Trait-level empathy**
Trait-level white lies	1.000		
Trait-level false beliefs	0.553^***^	1.000	
Trait-level empathy	0.425^**^	0.328^**^	1.000

^**^p < 0.01, ^***^p < 0.001.

#### 2.3.3 Effect of trait-level false beliefs and trait-level empathy on trait-level white lies among children with mild intellectual disabilities and typically developing children

Among children with mild intellectual disabilities, trait-level false beliefs (*t* = 2.444, *p* = 0.008 < 0.01) and trait-level empathy (*t* = 2.219, *p* = 0.017 < 0.05) positively predicted trait-level white lies. Among typically developing children, trait-level false beliefs (*t* = 3.447, *p* = 0.000 < 0.001) and trait-level empathy (*t* = 2.997, *p* = 0.002 < 0.01) positively predicted trait-level white lies (Table 4).

**Table 4:** Results of regression analysis of trait-level white lies, trait-level false beliefs, and trait-level empathy among children with mild intellectual disabilities and typically developing children.

	**Dependent variable**	**Independent variable**	**β**	***t***	***p***	**R2**	**Adjusted R^2^**
Children with mild intellectual disabilities	Trait-level white lies	Trait-level false beliefs	0.701	2.444^**^	0.008	0.223	0.286
		Trait-level empathy	0.698	2.219^*^	0.017		
Typically developing children	Trait-level white lies	Trait-level false beliefs	0.840	3.447^***^	0.000	0.260	0.341
		Trait-level empathy	0.725	2.997^**^	0.002		

^*^p < 0.05, ^**^p < 0.01, ^***^p < 0.001.

### 2.4 Discussion

The results of Experiment 1 confirm that the development level of trait-level false beliefs among children with mild intellectual disabilities is, to some degree, lower than that among typically developing children. Existing research on trait-level false beliefs has primarily focused on typically developing children. For example, used scales to explore the intricate tapestry of the development of trait-level false beliefs among typically developing children. Research on trait-level false beliefs among children with intellectual disabilities remains scant. Therefore, this study makes a crucial contribution to the literature and provides a foundation for future research on this enigmatic topic.

Similar to the findings concerning trait-level false beliefs, this study reveals that the development of trait-level empathy among children with mild intellectual disabilities lags behind that among typically developing children. Most studies on trait-level empathy, both domestic and international, have focused on typically developing children. For instance, Bryant (1982) used scales to examine the development of trait-level empathy among typically developing children. However, research on trait-level empathy and children with intellectual disabilities is scarce, creating a critical knowledge gap. This study makes a significant contribution to the literature by providing foundational data for future research on the development of trait-level empathy among children with intellectual disabilities.

The results of Experiment 1 also indicated that the development of trait-level white lies among children with mild intellectual disabilities is lower than that among typically developing children. Research on this captivating trait, similar to that on false beliefs and empathy, has primarily focused on typically developing children. For instance, Lundquist et al. (2009) and Hart et al. (2020) used scales and conducted elaborate studies on the developmental characteristics and levels of typically developing children. However, trait-level white lies remain underexplored among children with intellectual disabilities. This study explores the topic and lays the foundation for future research on the kaleidoscopic development of trait-level white lies among children with intellectual disabilities.

Finally, the results showed that trait-level false beliefs play a bigger role than trait-level empathy in shaping trait-level white lies among children with mild intellectual disabilities. Given the scarcity of research on trait-level false beliefs, empathy, and white lies among children with intellectual disabilities, this study provides salient results. By employing multiple linear regression analysis, this study transcends the traditional boundaries of research, reveals the complex relationship between the three constructs, and expands existing knowledge on trait-level white lies among children with mild intellectual disabilities. This contribution is not only captivating, but it also serves as the cornerstone of future research in this field.

## 3 Experiment 2: Effect of state-level false beliefs and state-level empathy on state-level white lies among children with mild intellectual disabilities

### 3.1 Objective and hypotheses

Experiment 2 tested Hypotheses 2 and 4. More specifically, it examined the influence of state-level false beliefs and state-level empathy on state-level white lies among children with mild intellectual disabilities and typically developing children. It investigated whether differences in state-level false beliefs and state-level empathy between the two groups of children lead to differences in their state-level white lies.

### 3.2 Methods

#### 3.2.1 Participants

The participants in Experiment 2 were the same as those in Experiment 1. As stated earlier, there were no significant differences in the verbal intelligence scores of the two groups of children (*z* = −1.52, *p* = 0.103).

#### 3.2.2 Experimental design

This study used a single-factor between-subjects design. The independent variable was the group of children. The dependent variables were the scores on the state-level white lies, state-level false beliefs, and state-level empathy tasks.

#### 3.2.3 Measures

##### 3.2.3.1 State-level white lies

A hide-and-seek task was performed to assess state-level white lies. First, the experimenter showed the photo of the participant's best friend and that of an unknown child to the participant. The experimenter introduced the unknown child, saying “Hello! Welcome to today's experiment. This is a child you don't know. His name is Lele. He is as old as you are and goes to kindergarten like you.” Then, the experimenter showed two paper cups cut in half and placed them in front of the participant. The open ends of the cups were facing the participant such that the participant could see what was inside the cups but the experimenter could not. Then, the participant was asked to put a toy deer in one of the cups. After this, the participant was asked if they could see which cup the toy was in. Upon receiving an affirmative response, the experimenter switched positions with the participant and asked the participant whether they could see the cup in which the toy was hidden. If the response was negative, the experimental setup was considered correct.

Next, the participant was asked to hide the toy deer in one of the cups, while the experimenter closed their eyes. Then, the experimenter told the participant, “I'll ask you which cup the toy is in, and you just point to the cup in which the toy is hidden. If I don't find the toy in the cup you point to, the toy will be given to your best friend or the unknown child. However, if I find the toy, the toy will be returned to the store.” Each situation (that is, the situation of the best friend or the unknown child) was repeated five times in a random order. In each attempt, if the participant pointed to an empty cup, they displayed a state-level white lie and scored one point. The total score of state-level white lies was the sum of the scores of the two situations (De La Cerda and Warnell, 2020; Chandler et al., 1989). The duration of this task was 5-10 minutes.

##### 3.2.3.2 State-level false beliefs

**Figure 4:**
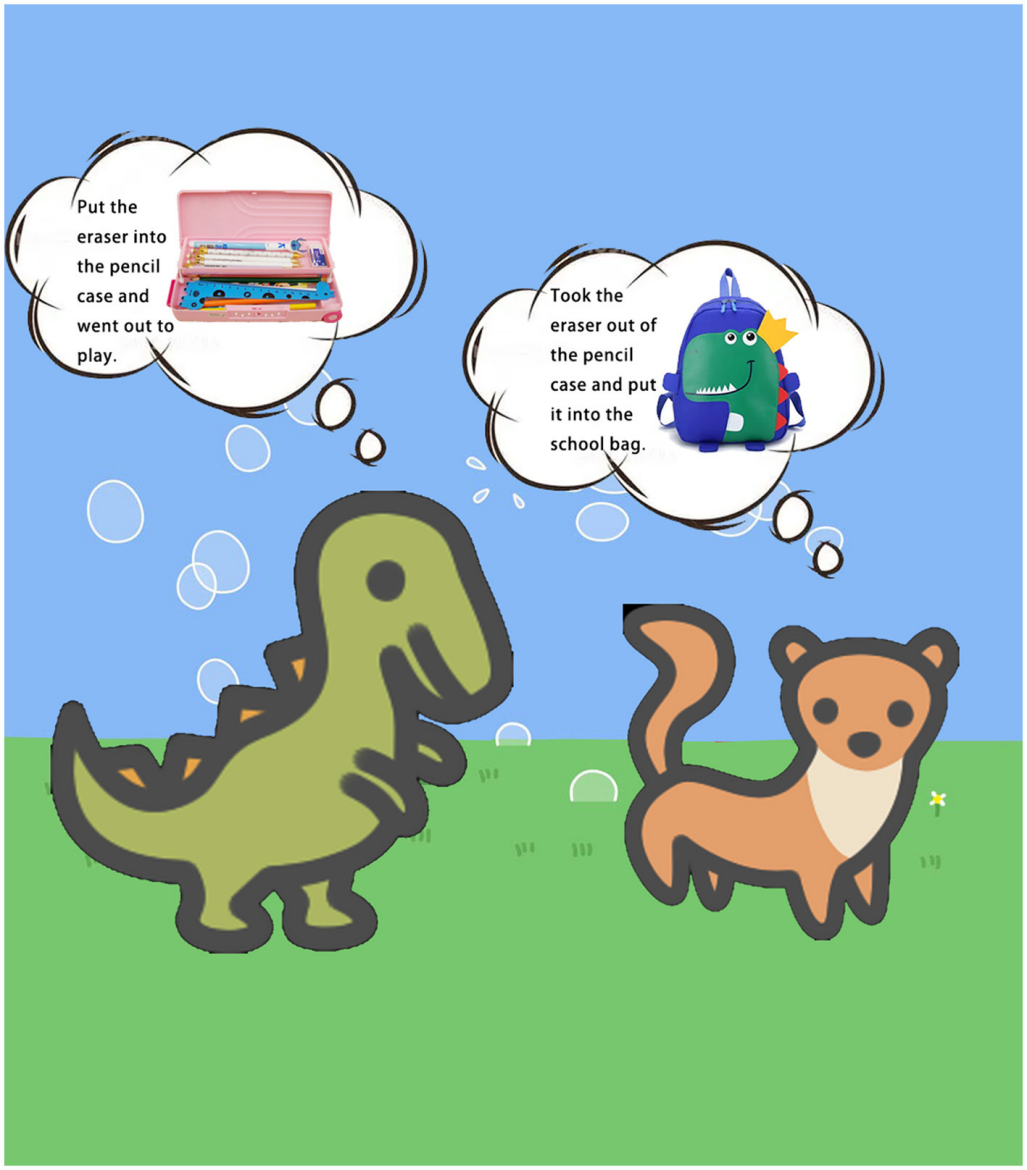
The story narrated in the unexpected location task to measure state-level false beliefs.

Two tasks were conducted to evaluate state-level false beliefs: the unexpected location task and the unexpected content task. In the unexpected location task, the experimenter placed a toy classroom, a toy pencil case, a toy eraser, a toy school bag, and a boy and a girl doll on an empty table and narrated a story: “A little girl Beibei and a little boy Lele were in the classroom. The bell rang, and BeiBei put the eraser in the pencil case and went out to play. Lele took the eraser out of the pencil case and put it in the school bag (Figure 4). After a while, class started, and Beibei returned to the classroom.” After narrating the story, the experimenter asked these questions from the participant: (1) “Where did Beibei put the eraser when she went out to play?” (2) “Do you know where the eraser is now?” (3) “Where will Beibei think the eraser is when she comes back to the classroom?” (4) “Where will Beibei look for the eraser?” One point each was awarded for correct responses to Questions 3 and 4 (Baron-Cohen et al., 1985). This task lasted 5-10 minutes.

**Figure 5:**
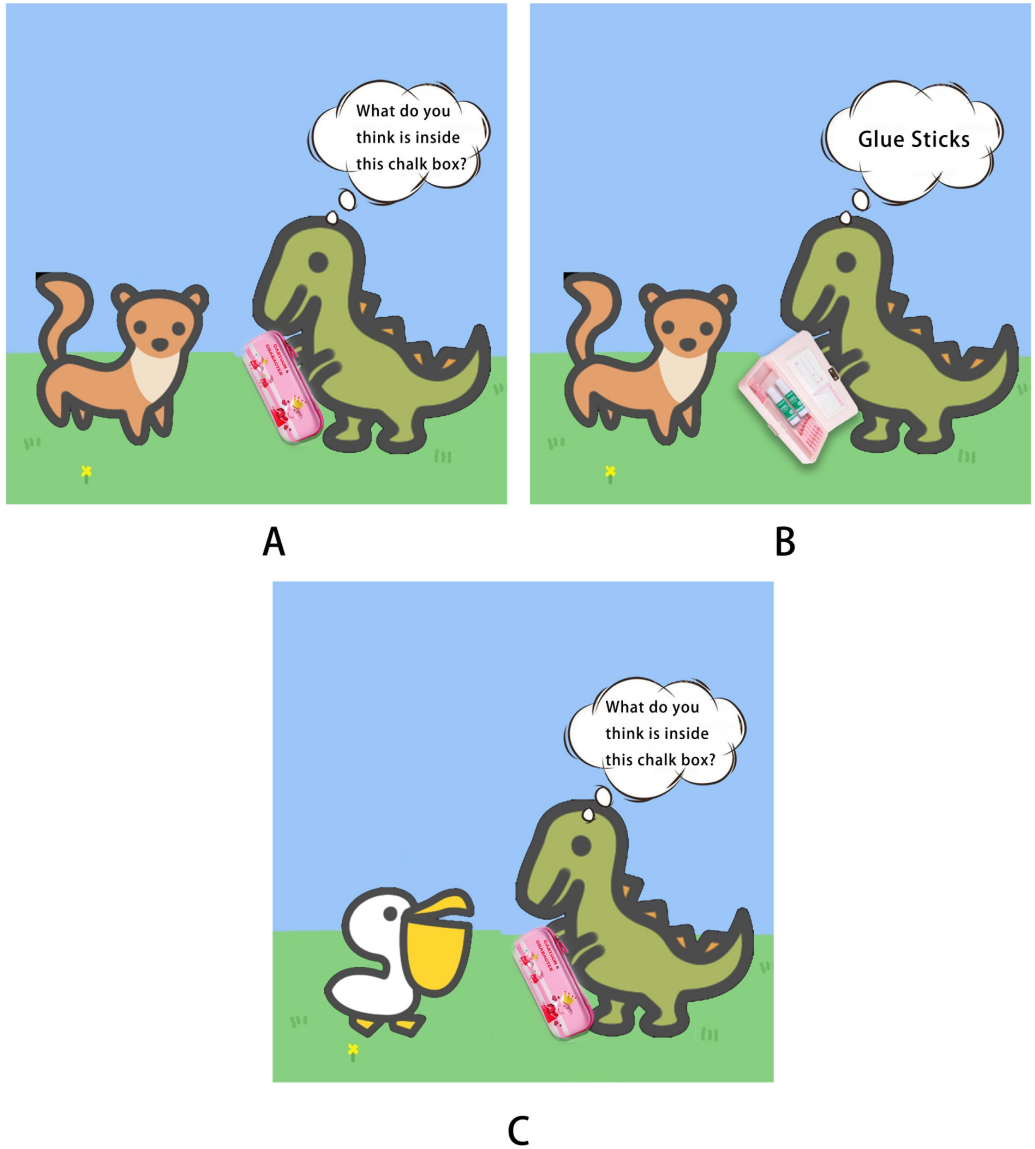
The story narrated in the unexpected content task to measure state-level false beliefs.

In the unexpected content task, there was a chalk box that contained glue sticks instead of chalks. The experimenter presented the chalk box to the participant and asked them to guess what was inside the box (Figure 5A). After receiving an answer, the experimenter opened the box to reveal the glue sticks (Figure 5B). Then, the experimenter closed the box and asked these questions from the participant: (1) “What is in the chalk box?” (2) “What would you think was inside the box if the lid was not open?” (3) “What did you think was inside the box before I opened the lid?” (4) “If your friend comes over and I don't open the lid of the box, what will they think is inside the box?” (Figure 5C). One point each was awarded for correct answers to Questions 3 and 4 (Baron-Cohen et al., 1985). These tasks lasted 5-10 minutes.

##### 3.2.3.3 State-level empathy

State-level empathy was evaluated using empathy stories, which were selected from Cole (1986). In this task, the experimenter presented cards depicting happy and sad emotions (e.g., crying and laughing faces) and observed the participant's reactions. Then, the experimenter told stories containing happy and sad feelings. Story 1 was a story containing happy feelings: “Beibei tidied up her room by herself, and her mother gave her a chocolate.” After narrating this story, the experimenter asked these questions from the participant: (1) “When Beibei's mother gave Beibei a chocolate, Beibei must have felt (a) happy (one point), (b) sad, or (c) not feel anything?” (2) “If you saw Beibei's mother give Beibei a chocolate, you would have felt (a) happy (one point), (b) sad, or (c) not feel anything?” (3) “Why are you like this?” (one point).

Story 2 contained sad feelings: “Fan Fan was behaving disobediently in the class, and the teacher criticized Fan Fan.” After narrating this story, the experimenter asked these questions from the participant: (1) “When the teacher criticized Fan Fan, Fan Fan must have felt (a) happy, (b) sad (one point), or (c) not feel anything?” (2) “If you see anyone being criticized, you will feel (a) happy, (b) sad (correct response), or (c) not feel anything?” (3) “Why are you like this? (one point). This task lasted 5–10 min.

#### 3.2.4 Experimental procedure

The tasks were administered to the participants in a random order to avoid the sequential effect of the tasks. The total duration of Experiment 2 was ~25 min. All tasks were conducted in classrooms with the researcher providing detailed instructions to ensure scientific assessments. Before administering the questionnaires, all participants were trained on how to complete the questionnaires correctly. Data collection lasted 15–20 min during student breaks, and participants were informed that their participation would be voluntary and anonymous.

#### 3.2.5 Data analysis

The Mann-Whitney U test was used to compare the verbal intelligence scores of the two groups. Independent sample *t*-tests were performed to investigate differences in the scores for state-level false beliefs, state-level empathy, and state-level white lies between the two groups. Spearman's correlation was used to analyze the relationship between state-level false beliefs, state-level empathy, and state-level white lies in the two groups. Finally, multiple linear regression analysis was conducted to examine the influence of state-level false beliefs and state-level empathy on state-level white lies among children with mild intellectual disabilities and typically developing children. State-level white lies was the dependent variable, and state-level false beliefs and state-level empathy were the independent variables. All statistical analyses were performed using SPSS version 26.0.

### 3.3 Results

#### 3.3.1 State-level white lies, state-level false beliefs, and state-level empathy among children with mild intellectual disabilities and typically developing children

**Figure 6:**
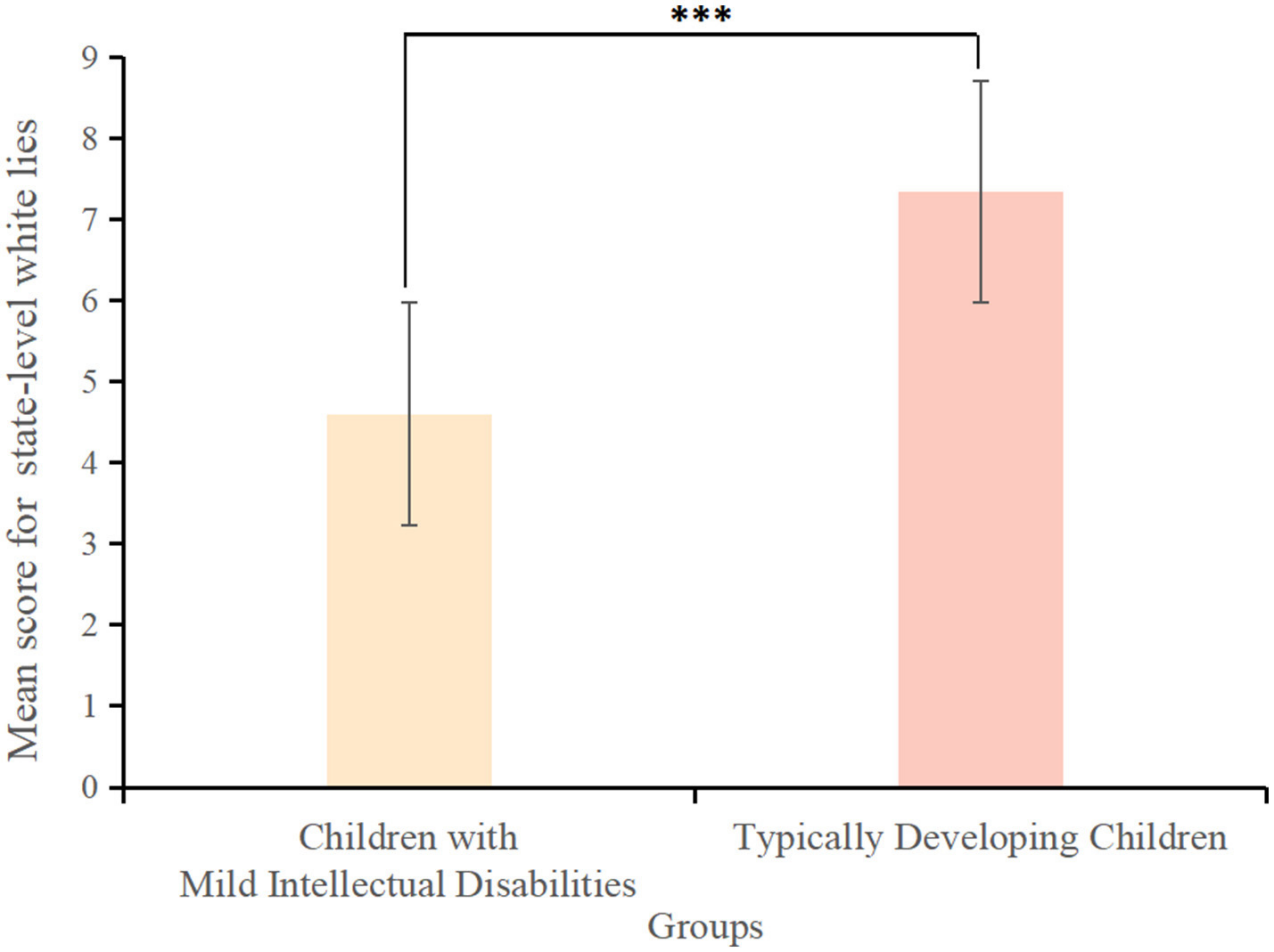
Scores for state-level white lies among children with mild intellectual disabilities and typically developing children. ***p < 0.001.

**Figure 7:**
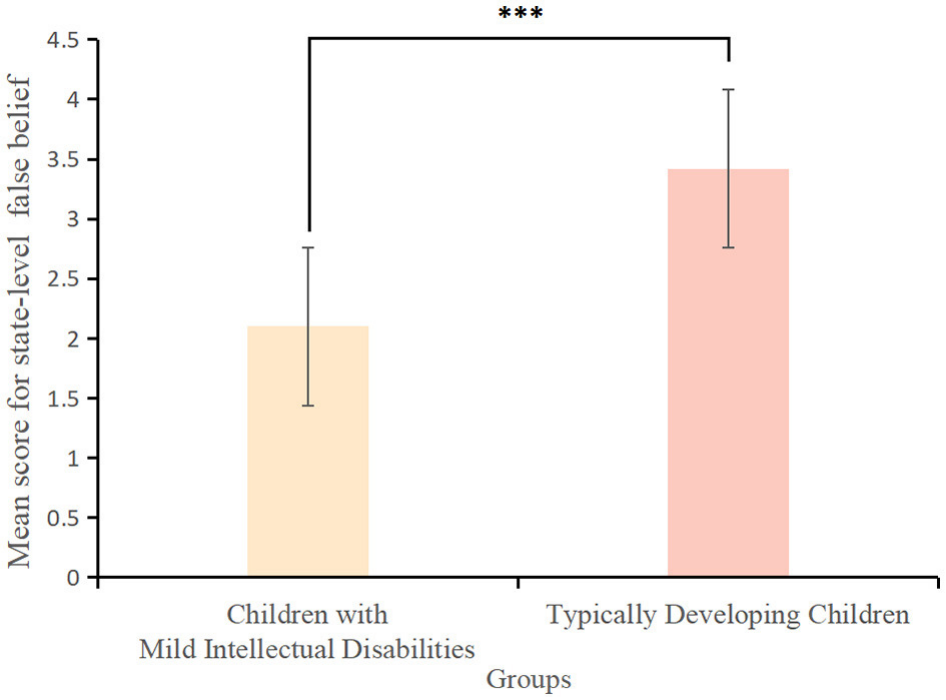
Scores for state-level false beliefs among children with mild intellectual disabilities and typically developing children. ***p < 0.001.

**Figure 8:**
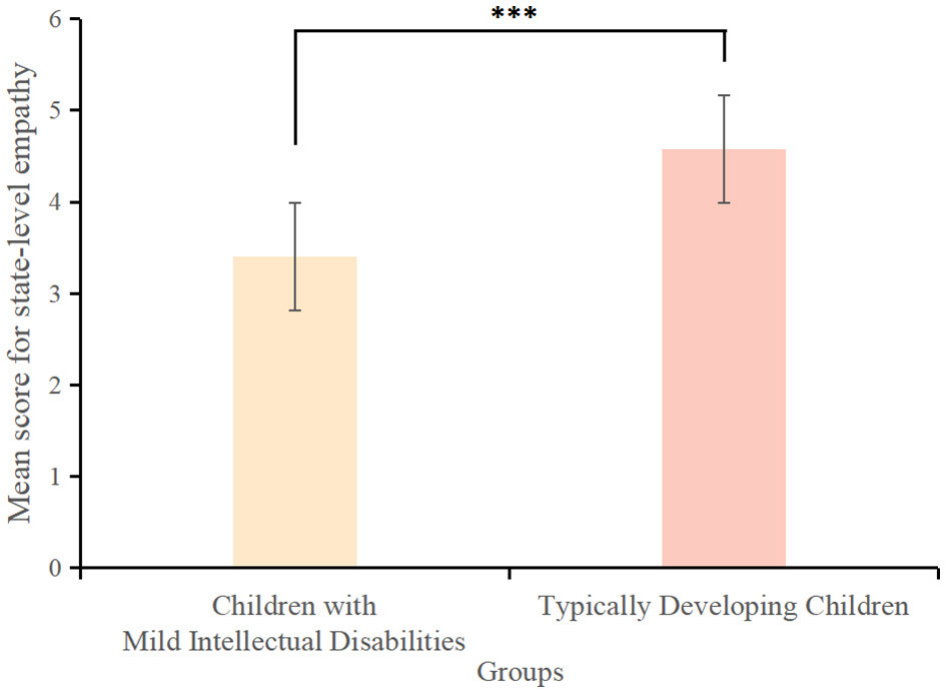
Scores for state-level empathy among children with mild intellectual disabilities and typically developing children. ***p < 0.001.

Children with mild intellectual disabilities scored significantly lower on state-level white lies (*M* ± *SD* = 4.60 ± 4.50) than typically developing children (*M* ± *SD* = 7.34 ± 3.10) {*t*_(163)_ = −3.87, *p* < 0.001, Cohen's *d* = −0.71, 95% *CI* = [−3.40, −1.48]} (Figure 6). They scored significantly lower on state-level false beliefs (*M* ± *SD* = 2.10 ± 1.47) than typically developing children (*M* ± *SD* = 3.42 ± 0.88) {*t*_(163)_ = −5.84, *p* < 0.001, Cohen's *d* = 0.25, 95% *CI* = [−1.72,−0.92]} (Figure 7). Similarly, they scored significantly lower on state-level empathy (*M* ± *SD* = 3.40 ± 1.80) compared to typically developing children (*M* ± *SD* = 4.58 ± 1.79) {*t*_(163)_ = −3.81, *p* < 0.001, Cohen's *d* = −0.66, 95% *CI* = [−1.75, −0.62]{ (Figure 8).

#### 3.3.2 Correlation between state-level white lies, state-level false beliefs, and state-level empathy among children with mild intellectual disabilities and typically developing children

Among children with mild intellectual disabilities, state-level white lies were significantly and positively correlated with state-level false beliefs (*r* = 0.42, *p* < 0.01) and state-level empathy (*r* = 0.22, *p* < 0.05). Additionally, state-level false beliefs and state-level empathy were significantly and positively correlated (*r* = 0.47, *p* < 0.01) (Table 5). State-level white lies were significantly and positively correlated with state-level false beliefs (*r* = 0.45, *p* < 0.01) and state-level empathy (*r* = 0.28, *p* < 0.05) among typically developing children also. State-level empathy and state-level false beliefs were also correlated (*r* = 0.42, *p* < 0.01) (Table 6).

**Table 5:** Results of correlation analysis of state-level white lies, state-level false beliefs, and state-level empathy among children with mild intellectual disabilities.

	**State-level white lies**	**State-level false beliefs**	**State-level empathy**
State-level white lies	1.00		
State-level false beliefs	0.42^**^	1.00	
State-level empathy	0.22^*^	0.47^**^	1.00

^*^p < 0.05, ^**^p < 0.01.

**Table 6:** Results of correlation analysis of state-level white lies, state-level false beliefs, and state-level empathy among typically developing children.

	**State-level white lies**	**State-level false beliefs**	**State-level empathy**
State-level white lies	1.00		
State-level false beliefs	0.45^**^	1.00	
State-level empathy	0.28^*^	0.42^**^	1.00

^*^p < 0.05, ^**^p < 0.01.

#### 3.3.3 Effect of state-level false beliefs and state-level empathy on state-level white lies among children with mild intellectual disabilities and typically developing children

Among children with mild intellectual disabilities, both state-level false beliefs (*t* = 3.12, *p* = 0.004 < 0.01) and state-level empathy (*t* = 2.74, *p* = 0.03 < 0.05) positively predicted state-level white lies. Similarly, state-level false beliefs (*t* = 3.38, *p* = 0.002 < 0.01) and state-level empathy (*t* = 2.87, *p* = 0.01 < 0.05) positively predicted state-level white lies among typically developing children (Table 7).

**Table 7:** Results of regression analysis of state-level white lies, state-level false beliefs, and state-level empathy among children with mild intellectual disabilities and typically developing children.

	**Dependent variable**	**Independent variable**	**β**	***t***	**R2**	**Adjusted R^2^**
Children with mild intellectual disabilities	State-level white lies	State-level false beliefs	0.56	3.12^**^	0.27	0.34
		State-level empathy	0.30	2.74^*^		
Typically developing children	State-level white lies	State-level false beliefs	0.55	3.38^**^	0.25	0.23
		State-level empathy	0.27	2.87^*^		

^*^p < 0.05, ^**^p < 0.01.

### 3.4 Discussion

Consistent with the findings of previous studies, Experiment 2 showed that the development of state-level false beliefs is lower among children with mild intellectual disabilities than among typically developing children. Explored differences in the development of state-level false beliefs among children with intellectual disabilities and typically developing children with similar developmental levels. They found that the development is significantly slower among children with mild intellectual disabilities than among typically developing children, but the developmental trends are generally the same in both groups. They also found that male and female children with intellectual disabilities exhibit similar levels of development. Peterson and Bowler (2000) also found that children with intellectual disabilities develop state-level false beliefs slower than typically developing children. Overall, this study confirms the findings of previous studies on state-level false beliefs in children with intellectual disabilities. More importantly, it extends the literature by revealing the applicability of existing measurement tools in this population, analyzing differences in state- and trait-level false beliefs, exploring the relationship between false beliefs and white lie behaviors, and providing a detailed analysis of individual developmental differences. The findings offer new perspectives and evidence for the theoretical deepening and methodological improvement of research on state-level false beliefs among children with intellectual disabilities.

Experiment 2 also showed that the development of state-level empathy is slower among children with mild intellectual disabilities than among typically developing children. This result aligns with the findings, who compared state-level empathy among children with autism (i.e., children with intellectual disabilities) and typically developing children with similar developmental levels. State-level empathy was lower among children with intellectual disabilities than among typically developing children. Our finding expands research on state-level empathy in children with intellectual disabilities by systematically comparing, for the first time, state- and trait-level empathy in this population. It clarifies the importance of state-level empathy in the social interactions of these children. Additionally, it reveals the salience of existing measurement tools in evaluating state-level empathy among children with low cognitive abilities. Overall, this study provides theoretical support for the development of state-level empathy among children with intellectual disabilities.

Finally, Experiment 2 revealed that, among the factors influencing state-level white lies in children with mild intellectual disabilities, state-level false beliefs play a primary role. Previous studies on state-level white lies have mostly explored the individual effects of state-level false beliefs and state-level empathy among typically developing children. They have not examined their combined effect. Investigated the developmental characteristics of state-level white lies in preschoolers by selecting the influencing factors closely related to state-level white lies, such as state-level false beliefs and state-level empathy. However, they did not examine the interactive influence of the two variables. Meiqin et al. (2022) conducted a similar study among individuals aged 3-10 years, improving the scope of the study. However, they also did not examine the combined effect of state-level false beliefs and state-level empathy. This study confirmed Hypothesis 3 by combining the two influencing factors through multiple linear regression analysis. Consequently, it not only verified their influence on state-level white lies among children with mild intellectual disabilities but also extended the literature on the determinants of state-level white lies among typically developing children.

## 4 General discussion

### 4.1 Differences in state- and trait-level false beliefs among children with mild intellectual disabilities

This study confirmed Hypotheses 1 and 3, revealing that children with mild intellectual disabilities show significantly slower development of both state- and trait-level false beliefs than typically developing children. Specifically, children with mild intellectual disabilities exhibit notable deficits in understanding others' mental states and intentions, particularly when they must infer and understand others' beliefs. This finding aligns with the results, as well as, who found that children with intellectual disabilities exhibit significant delays in social cognitive development.

Additionally, a unique contribution of this study is its in-depth analysis of the applicability of existing tools for measuring state-level false beliefs. This study found that tools designed to measure state-level false beliefs are more suitable for children with mild intellectual disabilities, emphasizing the importance of choosing appropriate measurement tools for evaluating such children. This finding provides valuable insight for improving data accuracy through better tool selection. Moreover, it supports the view of Hronis et al. (2017) that children with low cognitive abilities perform poorly on complex cognitive tasks and thus require tools with low cognitive demands (Hronis et al., 2017).

### 4.2 Differences in state- and trait-level empathy among children with mild intellectual disabilities

This study confirmed Hypotheses 2 and 4 by assessing state- and trait-level empathy among children with mild intellectual disabilities. Specifically, the results showed that children with mild intellectual disabilities demonstrate lower empathy than typically developing children. Although the differences between state- and trait-level empathy did not reach statistical significance, both forms of empathy showed developmental delays. This result is consistent with the findings and further supports the notion that children with mild intellectual disabilities struggle with emotional understanding and responses.

It is worth noting that this study innovatively analyzed the impact of measurement tools on the assessment of empathy. Through a detailed analysis of questionnaires and scales, this study revealed that the choice of measurement tool significantly affects evaluation outcomes among children with low cognitive abilities. Certain tools may be better suited for assessing empathy among these children, whereas others may be inappropriate because of their complexity or high cognitive load. This finding corroborates the findings of Kruit et al. (2018) and validates the critical role of measurement tools in cognitive ability assessments (Kruit et al., 2018).

### 4.3 Influence of false beliefs and empathy on white lie behavior: behavioral prediction based on multi-factor analysis

This study revealed a significant and positive predictive effect of false beliefs on white lie behavior. This finding is consistent with those of Roby and Scott (2016) and Scott and Baillargeon, 2017 and confirms the importance of false beliefs in children's white lie behavior. Specifically, the results showed that the greater the development of false beliefs, the more frequent the white lie behavior among children with mild intellectual disabilities, suggesting that false beliefs play a key role in predicting children's social behavior.

Another contribution of this study is that it reveals that false beliefs are a bigger predictor of white lie behavior than empathy. While empathy is a crucial determinant of children's social behavior, this study demonstrates that false beliefs predict white lie behavior more strongly. This result aligns with previous findings suggesting that false beliefs play a crucial role in children's social cognitive development (Roby and Scott, 2016; Scott and Baillargeon, 2017). Through a combined analysis of false beliefs and empathy, this study deepens our understanding of the social cognitive development of children with mild intellectual disabilities and broadens the perspective on the mechanisms affecting social cognition disorders.

### 4.4 Limitations and directions for future research

Although this study has salient findings, it has several limitations. First, the selection of the study sample may have limited the generalizability of the results. As the participants were from specific regions or educational environments, the findings may not be fully applicable to all children with mild intellectual disabilities. Future studies should recruit a more diverse sample by including children from different regions, cultures, and educational environments to enhance the external validity of the results. Second, although this study made innovative attempts in the selection of measurement tools, cognitive burden may still arise in practical applications. For example, some tools may be too complex to measure the cognitive abilities of children with mild intellectual disabilities. Future studies should focus on optimizing the design of these tools to ensure their suitability for children with low cognitive abilities. Third, this study examined the performance of children with mild intellectual disabilities in specific areas of social cognition (false beliefs and empathy). Future studies should examine other cognitive domains, such as executive function, language ability, and attention control, to gain a comprehensive understanding of the cognitive developmental characteristics of these children. This would also help develop comprehensive educational programs and interventions for children with mild intellectual disabilities.

## 5 Conclusion

This study reveals the developmental characteristics of state- and trait-level false beliefs, empathy, and white lies among children with mild intellectual disabilities. In particular, it shows that, compared with typically developing children, children with mild intellectual disabilities exhibit lower trait-level false beliefs, empathy, and white lies. Trait-level false beliefs and empathy positively predict trait-level white lies among children with mild intellectual disabilities. Children with mild intellectual disabilities also exhibit lower state-level false beliefs, empathy, and white lies than typically developing children. Furthermore, state-level false beliefs and empathy positively predict state-level white lies among children with mild intellectual disabilities. Overall, this study extends the existing literature and provides new measurement tools and methodological guidance for future research. The findings of this study have practical implications for the development of educational and intervention strategies for children with mild intellectual disabilities.

## Data Availability

The original contributions presented in the study are included in the article/supplementary material, further inquiries can be directed to the corresponding author.
